# Improving memory following prefrontal cortex damage with the PQRST method

**DOI:** 10.3389/fnbeh.2015.00211

**Published:** 2015-08-12

**Authors:** Elisa Ciaramelli, Francesco Neri, Luca Marini, Davide Braghittoni

**Affiliations:** ^1^Dipartimento di Psicologia, Università di BolognaBologna, Italy; ^2^Centro di Studi e Ricerche in Neuroscienze Cognitive, CesenaItaly

**Keywords:** long-term memory, episodic memory, cognitive rehabilitation, prefrontal cortex, amnesia

## Abstract

We tested (1) whether the PQRST method, involving Preview (P), Question (Q), Read (R), State (S), and Test (T) phases, is effective in enhancing long-term memory in patients with mild memory problems due to prefrontal cortex lesions, and (2) whether patients also benefit from a more self-initiated version of the PQRST. Seven patients with prefrontal lesions encoded new texts under three different conditions: the Standard condition, requiring to read texts repeatedly, the PQRST-Other condition, in which the experimenter formulated questions about the text (Q phase), and the PQRST-Self condition, in which patients formulated the relevant questions on their own. Compared to the Standard condition, both the PQRST-Other and the PQRST-Self condition resulted in higher immediate and delayed recall rates, as well as a higher ability to answer questions about the texts. Importantly, the two PQRST conditions did not differ in efficacy. These results confirm that the PQRST method is effective in improving learning of new material in brain-injured populations with mild memory problems. Moreover, they indicate that the PQRST proves effective even under conditions with higher demands on patients’ autonomy and self-initiation, which encourages its application to real-life situations.

## Introduction

Long-term memory disorders are among the most challenging cognitive impairments following acquired brain damage, and may have a profound impact on patients’ daily living, ranging from minimal forgetfulness to a pervasive inability to learn new information and cope with life demands. For this reason, there is great interest in developing methods aimed at restoring or compensating memory impairment.

Memory is a multicomponential process. Consequently, memory deficits are multifaceted, and call for different treatment options. Lesions to the medial temporal lobe may result in an inability to explicitly encode and retrieve new information, while implicit memory is spared (Kopelman, 2002; Wilson, 2009). Damage to the prefrontal cortex more commonly hampers “working-with-memory” processes supporting encoding and retrieval operations (Moscovitch, 1992; Shimamura, 1995). Prefrontal cortex may assist encoding operations by favoring selection of goal-relevant incoming information (Otten et al., 2001; Badre and Wagner, 2007; Blumenfeld and Ranganath, 2007) and its meaningful organization in working memory (Fletcher and Henson, 2001; Blumenfeld and Ranganath, 2007). At retrieval, prefrontal cortex may support selection of relevant memories according to retrieval goals (Kuhl et al., 2007; Barredo et al., 2015) and monitoring processes assessing the veridicality of the retrieval output (Gilboa and Moscovitch, 2002; Ciaramelli and Spaniol, 2009).

While treatment options for severe amnesia include techniques based on spared implicit memory processes and memory aids (Baddeley and Wilson, 1994; Wilson, 2009; Ptak et al., 2010), patients with less severe deficits and partial sparing of explicit memory may benefit from “internal” mnemonics directed at optimizing encoding and retrieval operations (Van der Linden and Van der Kaa, 1989; Ptak et al., 2010). These include the generation of images connected to the material to be learned (e.g., Jones, 1974; Kaschel et al., 2002), verbal strategies (Harris, 1992), and optimal structuring of information (Doornheim and De Haan, 1998). The PQRST method is one such strategy (Moffat, 1992).

PQRST is the acronym of *Preview* (establish the general theme of the text), *Question* (formulate main questions about the text), *Read* (read carefully, thinking at the questions), *State* (summarize the main information), and *Test* (test your knowledge) (Moffat, 1992). This method drives individuals through an ordinated series of steps favoring deep analysis and organization of texts to be learned, which improves later retention (Wilson, 2009).

Wilson (1987, 2009) has tested the efficacy of PQRST empirically in several cases of memory-impaired patients. First, she described a few cases of patients with severe amnesia due to various etiologies who studied newspaper articles either under PQRST instructions or repeated practice instructions (i.e., they were read the passages several times). Compared to repeated practice, PQRST generally led to an improvement in answering questions about the text. Since questions were part of the PQRST learning procedure, however, the observed improvement may be due to “encoding specificity,” the reinstatement of the original learning situation at recall (Tulving and Thomson, 1973). Patients, indeed, did not improve in free recall, suggesting that PQRST brings little benefit to patients with severe amnesia (Wilson, 1987). Different seems the case of patients with less severe memory deficits. In a group of patients with mild memory deficits due to traumatic brain injury (TBI), the PQRST method led to an improvement not only in answering questions about the texts, but also in (delayed) recall (Wilson, 1987). In this case, the improvement could not be explained by encoding specificity alone. Rather, the PQRST seems to optimize encoding operations, though it is not completely clear through which mechanisms. One candidate mechanism is deep encoding. According to Craik and Lockhart (1972), material processed at a deep (e.g., semantic) level is retained better than information processed at a shallow (e.g., phonological) level, and PQRST (compared to repeated practice) demands a deeper semantic analysis of the text in order to answer the relevant questions. More recently, Franzen et al. (1996) have applied the PQRST to two young TBI patients. Both patients improved following the PQRST compared to a control “metacognitive” treatment, but returned to baseline levels soon (Franzen et al., 1996). As well, Bussman-Mork et al. (2000) noted that PQRST led to better retention of new material compared to no-treatment, but that it was lacking in generalization (Wilson, 2009). In sum, there is some evidence that the PQRST method is effective in improving long-term memory in patients with mild memory deficits, but there are not many studies evaluating its efficacy. Moreover, extant studies raise concerns about the generalization of training effects.

The first aim of this study is to test the efficacy of the PQRST method in a group of patients with mild memory problems due prefrontal cortex lesions. Given that prefrontal cortex supports strategic encoding and retrieval processes (Moscovitch, 1992), and that patients with prefrontal lesions may fail to adopt strategies spontaneously (Stuss et al., 1994; Gershberg and Shimamura, 1995; Alexander et al., 2009), we predict that this patient population should benefit enormously from strategic encoding conditions such as those promoted by the PQRST method. One second aim of the study pertains to the “Question phase” of the PQRST, which constitutes the “skeleton” around which the PQRST procedure unfolds. In principle, questions about the main aspects of the text may be formulated by the experimenter or the patients themselves. Previous studies have mainly adopted questions formulated by the experimenter, and no study so far has investigated whether the two strategies are equally effective. Of course, severely compromised patients may not be able to formulate questions on their own. However, if they are, one may predict an even larger effect of PQRST using self-generated questions, compared to other-generated question. Self-generated questions would reflect those aspects of the text that captured patients’ attention, and would therefore be ideally suited to motivate patients to scrutinize the texts further. Recall, indeed, tends to be better for information that is more personally salient (e.g., Westmacott and Moscovitch, 2003). Understanding the effectiveness of self-generated questions would be important with respect to generalization. Generalization, that is, the spontaneous transfer of a trained technique to new material and real-life situations, is the ultimate goal of cognitive rehabilitation. One prerequisite for the spontaneous use of the PQRST in real life is the ability, on the patients’ part, to formulate questions on their own. Demonstrating that the PQRST method also works with self-generated questions, therefore, would be one first step toward promoting its use in real life.

To these aims, a group of patients with prefrontal lesions memorized texts in three different encoding conditions: a Standard condition, requiring to read the text repeatedly (see below), and two different PQRST conditions; in one, the questions were created by the experimenter (PQRST-Other condition), whereas in the other questions were created by the patients themselves (PQRST-Self condition). To foreshadow the results, we found that, compared to the Standard condition, both the PQRST-Other condition and the PQRST-Self condition resulted in better memory for the texts. Importantly, the PQRST-Self condition proved as effective as the PQRST-Other condition.

## Materials and Methods

### Participants

Participants were seven patients (one female) with lesions to the prefrontal cortex due to anterior communicating artery (AcoA) aneurysm or TBI (see **Table 1** for demographic and clinical data). Patients had a mean age of 45 years (range 32–60), and a mean education of 11.57 years (range 8–13). Time since injury was, on average, 7.4 years (range 1–20). Patients were recruited at the Centre for Studies and Research in Cognitive Neuroscience of the University of Bologna, in the context of a routine neuropsychological assessment, which highlighted, in all cases, long-term memory deficits. All patients complained about memory deficits in real life, and participated voluntarily to the study. Included patients were not receiving psychoactive drugs, and had no other diagnosis likely to affect cognition or interfere with the participation in the study (e.g., significant psychiatric disease, alcohol abuse, history of cerebrovascular disease) as determined by history. Participants gave written informed consent to participate in the study according to the Declaration of Helsinki (International Committee of Medical Journal Editors, 1991) and the Ethical Committee of the Department of Psychology, University of Bologna.

**Table 1:** Patients’ demographic and clinical data.

Patients:	C.C.	G.V.	A.B.	S.S.	C.2.	E.L.	V.2.	Mean
Age	41	52	60	51	32	31	48	45.0
Education	13	8	13	8	13	13	13	11.6
Gender	M	M	M	M	M	M	F	
Time since lesion (years)	1	7	6	1	13	4	20	7.4
Etiology	AcoA aneurysm	AcoA aneurysm	AcoA aneurysm	AcoA aneurysm	AcoA aneurysm	TBI	TBI	
Brain Damage	Bilateral vmPFC	Bilateral vmPFC, more pronounced on the right	Bilateral vmPFC	Bilateral vmPFC	Bilateral vmPFC	Bilateral frontal and temporal poles	Left prefrontal cortex	
MMSE	28	24	24	28	30	25	28	26.7
Attentional matrices	43 (2)	51 (4)	47 (3)	53 (4)	38 (2)	48 (3)	44 (3)	46.3 (3)
Stroop test -Errors (raw score)	1	0	0	0	0	0	0	0.1
WCST -Perseverative errors (%) (in percentile)	36 (1)	47 (1)	20 (30)	57 (1)	1 (50)	29 (1)	4 (50)	27.7 (19.1)
Phonemic fluency	18 (1)	22 (1)	38 (4)	29 (3)	27 (3)	19 (1)	29 (3)	26.0 (2.2)
Semantic fluency	30 (2)	40.5 (4)	52 (4)	48 (4)	58 (4)	29 (1)	63 (4)	45.7 (3.2)
Corsi test	3.5 (1)	4.5 (3)	3.75 (1)	6 (4)	3.5 (1)	4.5 (3)	5.75 (4)	4.5 (2.4)
Digit span	4.5 (2)	5 (3)	4.75 (4)	6 (4)	5.25 (4)	5.5 (4)	4.5 (2)	5.0 (3.2)
Word-list learning* -Immediate	0	0	1	3	3	0	0	1
-Delayed*	2	0	0	0	0	0	0	0.28
Prose passage recall task*	2	0	1	1	0	1	2	1
WMS	101	79	92	84	77	83	96	87.4

F, female; M, male; ACoA, anterior communicating artery; TBI, traumatic brain injury; WMS, Weschler Memory Scale. Unless noted, we report corrected scores, with the corresponding equivalent score (ES) in parentheses. *in these cases, we report ES only, because different versions of the test were used across patients, and therefore corrected scores are not comparable.

#### Neuropsychological Profile

Patients’ general cognitive functioning was generally preserved, as indicated by the scores they obtained in the Mini Mental State Examination, which were within the normal range in all cases (*M* = 26.7). Patients performed normally also in several tests assessing attentional and executive functions, such as selective attention (assessed with the Attentional Matrices test; Equivalent score (ES) = 3. Note that the ES ranges from 0 = impaired performance, and 1 = borderline performance, to 2–4 indicating normal performance; Spinnler and Tognoni, 1987), inhibition of automatic responses (assessed with the Stroop Color–Word test; mean number of errors = 0.14, cut off >7.5), semantic fluency (ES = 3.2; Spinnler and Tognoni, 1987) and phonemic fluency (ES = 2.2; Spinnler and Tognoni, 1987). Patients showed a weak performance in the Wisconsin Card Sorting Test, which was characterized by several perseverative errors (mean percentile score = 19.1). All patients, however, exhibited long-term memory deficits (Spinnler and Tognoni, 1987). On the Wechsler Memory Scale (Wechsler, 1945; Barletta-Rodolfi et al., 2011), patients’ mean general memory index was borderline (*M* = 87.4). Performance in immediate recall of word lists (assessed either with the Buschke–Fuld Test or the Rey 15 words test; Barletta-Rodolfi et al., 2011) and of a prose-passage recall task was weak (ES = 1 in both cases), and delayed recall of word lists was highly pathological (ES = 0.28; Spinnler and Tognoni, 1987). In contrast, scores in verbal short-term memory (ES = 3.2) and spatial short-term memory (ES = 2.4; assessed with Digit Span and Corsi test, respectively) were normal (Spinnler and Tognoni, 1987).

### Materials

Twenty-four prose passages were selected and adapted from various online media (e.g., online newspapers) as well as the reading comprehension section of a high school book. Each passage was between 145 and 190 words in length (*M* = 172, SD = 13), covered a single topic, and was divided into 28–30 idea units for scoring purposes. For each passage, four questions were developed, covering the main aspects of the story (other-generated questions; see below). The 24 prose passages were randomly divided into three sets of eight passages, matched for number of words [*F*(2,21) = 0.47; *p* = 0.62] and units [*F*(2,21) = 0.24; *p* = 0.78]. The assignment of the three sets to the different experimental conditions (Standard, PQRST-Other, PQRST-Self) was counterbalanced across participants.

### Procedures

The 24 prose passages were administered in 24 different experimental sessions. The alternance of experimental conditions across sessions was counterbalanced across participants (e.g., day 1: PQRST-Self; day 2: Standard; day 3: PQRST-Other: day 4: PQRST-Self, and so on), and the order of administration of each prose passage within each set was determined randomly for each participant. Depending on the experimental condition, participants received different encoding instructions.

#### PQRST-Other Condition and PQRST-Self Condition

In the Preview (P) phase, the experimenter read the passage aloud, to make the participants get a general idea of the material. The Question (Q) phase was different in the PQRST-Other and PQRST-Self conditions: in the PQRST-Other condition, the experimenter read the four (other-generated) questions about the text (e.g., How did the fireman solve the problem of the five people?). In the PQRST-Self condition, the patient formulated four questions regarding the text (self-generated questions). The experimenter stressed the need to formulate four questions that covered the whole story. In both PQRST conditions, the four questions were written on a card that was placed on the desk, in front of the participant, and remained there throughout the Read phase (R), in which participants read the material carefully to look for the answers to the questions. In the following State (S) phase, patients stated the answers, and, if necessary, read the text again. The whole “study session” lasted 10 min on average. Immediately afterward, the Test (T) phase began, in which the experimenter tested memory for the text by (1) asking the same questions that had been embedded in the study phase (i.e., the other-generated questions in the PQRST-Other condition, and the self-generated questions in the PQRST-Self condition), (2) asking for free recall of the passage, and (3) asking for delayed free recall of the passage, after 10 min of non-interfering activities (e.g., videogames). In the PQRST-Self condition, after delayed recall of the self-generated questions, patients were also asked the other-generated questions, i.e., those commonly used in the PQRST-Other condition. This was done to verify whether the improvement in answering questions about a text was limited to those questions that were part of the procedure, or generalized to untrained questions.

#### Standard Condition

The standard condition was designed to (1) be representative of patients’ usual encoding strategies and (2) last as long as the PQRST-based conditions. A preliminary, informal interview with each patient and a relative revealed that patients’ most common strategy to learn new material was to re-read texts over and over again, and in some cases highlight the relevant parts. In a second session, the experimenter asked patients to memorize a text, which confirmed that this was indeed the most frequent learning strategy they adopted. The Standard enconding condition was designed to mimic patients’ spontaneous strategies. First, similar to the two PQRST conditions, the experimenter read the passage aloud (Preview phase). Patients were then left free to read the text over and over again for 10 min, during which they could take notes and underline the most important parts (Study phase). Importantly, also in the Standard condition patients had the four questions about the passage introduced early on, which remained in front of them for the entire duration of the Study phase (see also Wilson, 1987). Patients were told that those questions highlighted the principal parts of the passage. This was done to verify if the mere availability of the four relevant questions accounted for performance improvements in the two PQRST conditions. After the study phase, patients were tested for retention in the same way as in the PQRST conditions (Test phase).

### Scoring

A scorer blind to the aim of the study and to the experimental hypotheses evaluated, for each prose passage, the frequency of passage units recalled correctly and the frequency of questions answered correctly. The scorer was instructed to consider an answer, or a passage unit, correct when it conveyed the relevant information completely and unambiguously, no matter whether verbatim or not.

## Results

Six dependent variables were considered: the frequency of passage units recalled immediately after the study phase and after the delay, and the frequency of correct answers given to the four questions, both immediately and after the delay. For the PQRST-Self condition, we also evaluated the frequency of correct answers given to the other-generated questions. In all cases, the variables were distributed normally (Komolgorov–Smirnov *p* > 0.20 in all cases) and were analyzed with parametric tests.

### Free Recall

**FIGURE 1:**
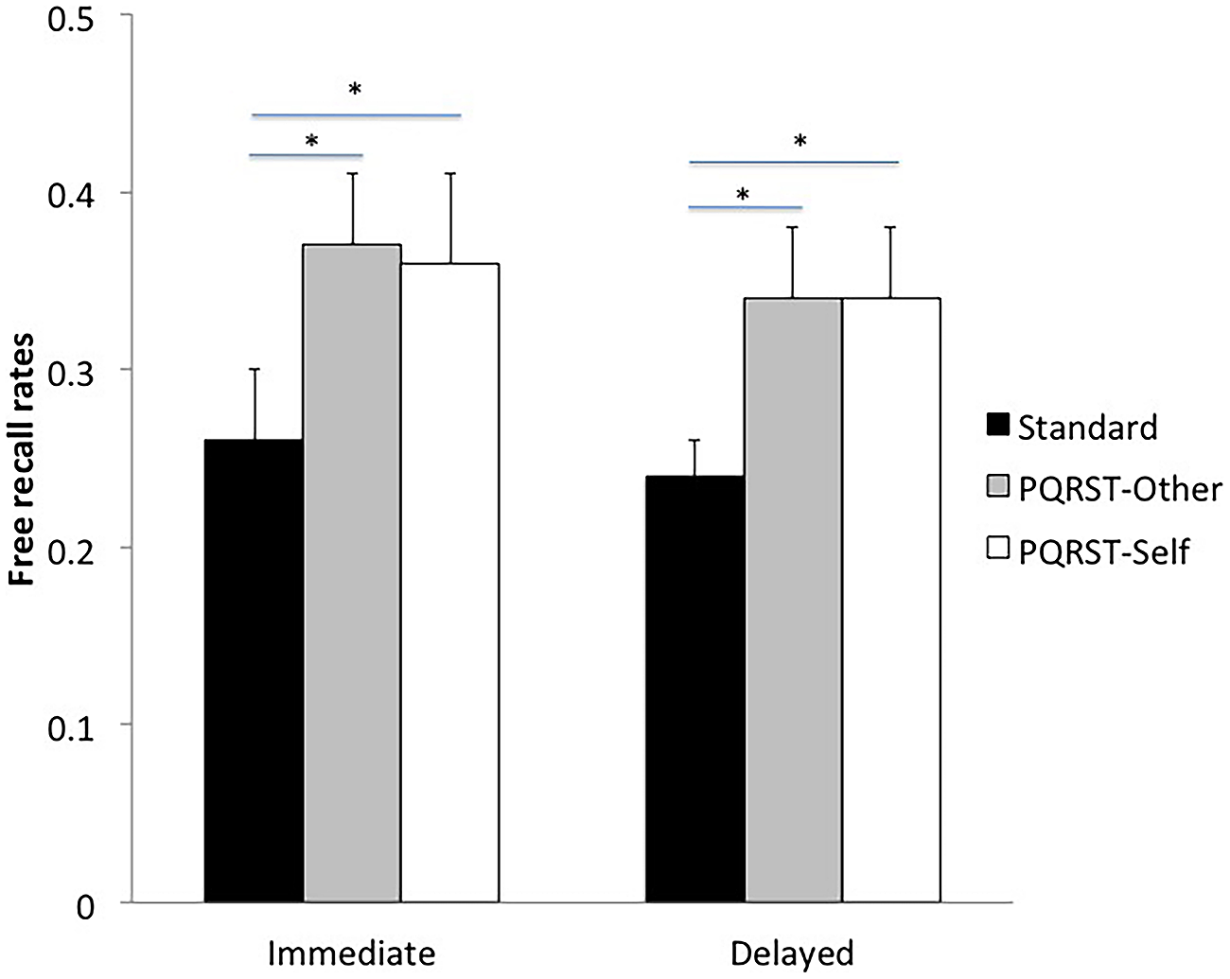
**Free recall rates by encoding condition (Standard, PQRST-Other, PQRST-Self) and test (immediate, delayed).** Bars represent the SE of the mean. *p < 0.05.

An analysis of variance (ANOVA) on free recall rates with Test (immediate, delayed) and Condition (PQRST-Other, PQRST-Self, Standard) as within-subject factors showed a significant effect of Test [*F*(1,6) = 11.72, *p* = 0.01], such that participants recalled more units at the immediate compared to the delayed test, and a significant effect of Condition [*F*(2,12) = 7.71, *p* = 0.007]. *Post hoc* Newmann–keuls tests showed that patients recalled more units in the PQRST-Other condition and in the PQRST-Self condition compared to the Standard condition (*p* < 0.05 in both cases), with no difference between the two PQRST conditions (*p* = 0.95; see **Figure 1**). The Condition × Test interaction was not significant (*p* = 0.82).

### Frequency of Correct Answers

**FIGURE 2:**
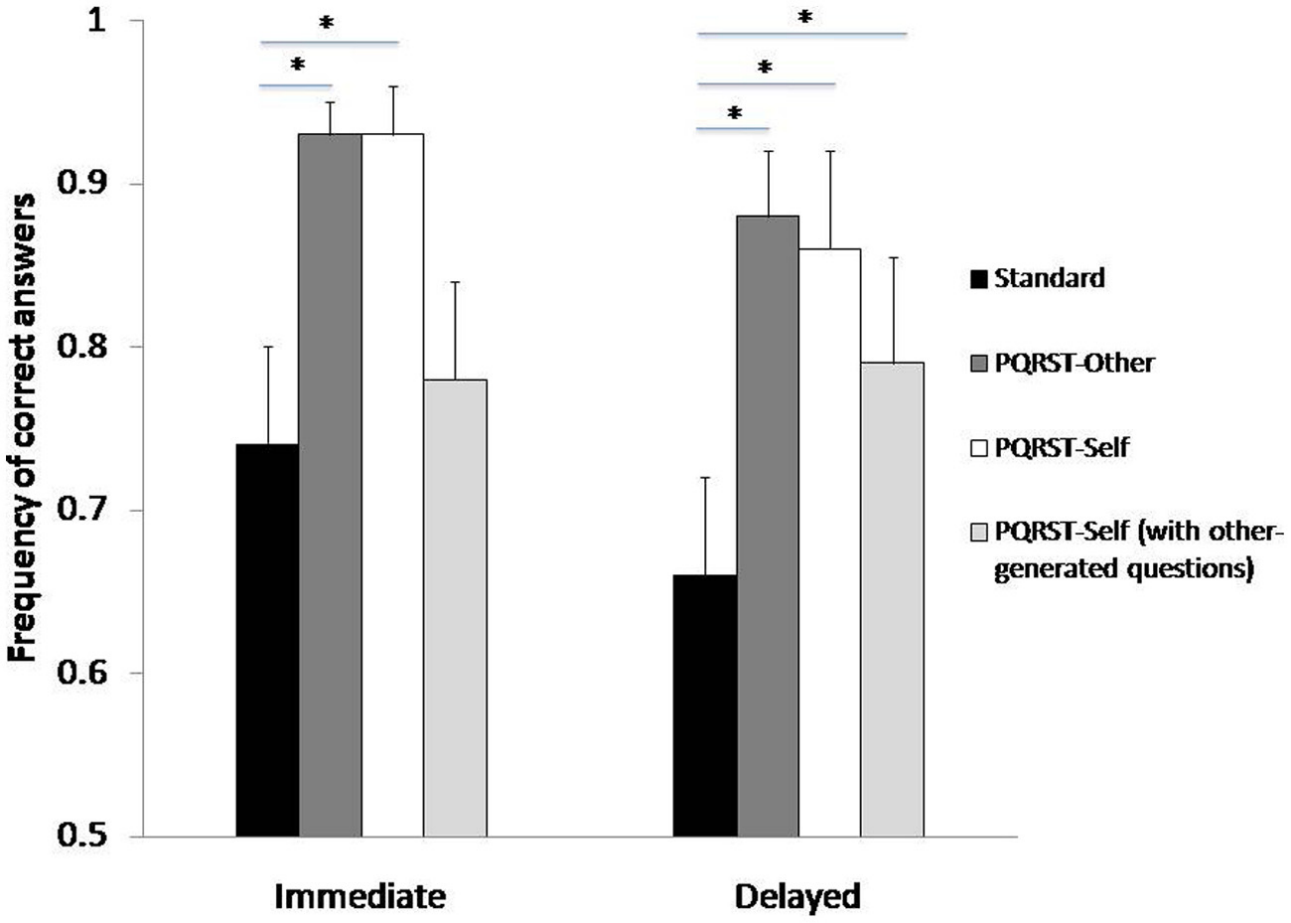
**Frequency of correct answers by encoding condition (Standard, PQRST-Other, PQRST-Self) and test (immediate, delayed).** Bars represent the SE of the mean. *p < 0.05.

Similar results were found on the ability to answer questions about the texts. The ANOVA on the frequency of correct answers with Test and Condition as factors showed a significant effect of Test [*F*(1,6) = 6.88, *p* = 0.04], such that correct answers were more frequent immediately after study than after a delay, and a significant effect of Condition [*F*(2,12) = 27.73, *p* = 0.000032]. *Post hoc* tests showed that patients answered more questions in the PQRST-Other condition and in the PQRST-Self condition compared to the Standard condition (*p* < 0.0003 in both cases), with no difference between the two PQRST conditions (*p* = 0.79) (see **Figure 2**). The Condition × Test interaction was not significant (*p* = 0.57).

We also ran the same ANOVA using the frequency of correct answers to the other-generated questions also for the PQRST-Self condition. There was a significant effect of Condition [*F*(2,12) = 15.65, *p* = 0.0005], which was qualified by a Condition X Test interaction [*F*(2,12) = 5.62, *p* = 0.02]. *Post hoc* tests showed that in the immediate testing session correct answers were more frequent in the PQRST-Other condition compared to the Standard condition (*p* < 0.0001), but there was no difference between the PQRST-Self and the Standard conditions (*p* = 0.15) (see **Figure 2**). However, in the delayed testing session, correct answers were more frequent in both the PQRST-Other and the PQRST-self condition compared to the Standard condition (*p* < 0.0004 in both cases) (see **Figure 2**). The PQRST-Other condition proved more effective than the PQRST-Self condition both immediately and after a delay (*p* < 0.006 in both cases).

### Neuropsychological Profile and PQRST Efficacy

In order to shed light on the type of patients that would benefit the most from the PQRST method, and that would benefit differentially from the classic, PQRST-Other method vs. the PQRST-Self method, we ran correlation analyses between patients’ results at standard neuropsycological tests and (1) the difference between scores attained in the PQRST-Other condition and the Standard condition, and (2) the difference between scores attained in the PQRST-Other condition and the PQRST-Self condition (in both cases collapsing across immediate and delayed conditions). Given that, in some cases, the variables were non-normally distributed (Komolgorov–Smirnov *p* < 0.05), we run non-parametric correlation analyses.

**FIGURE 3:**
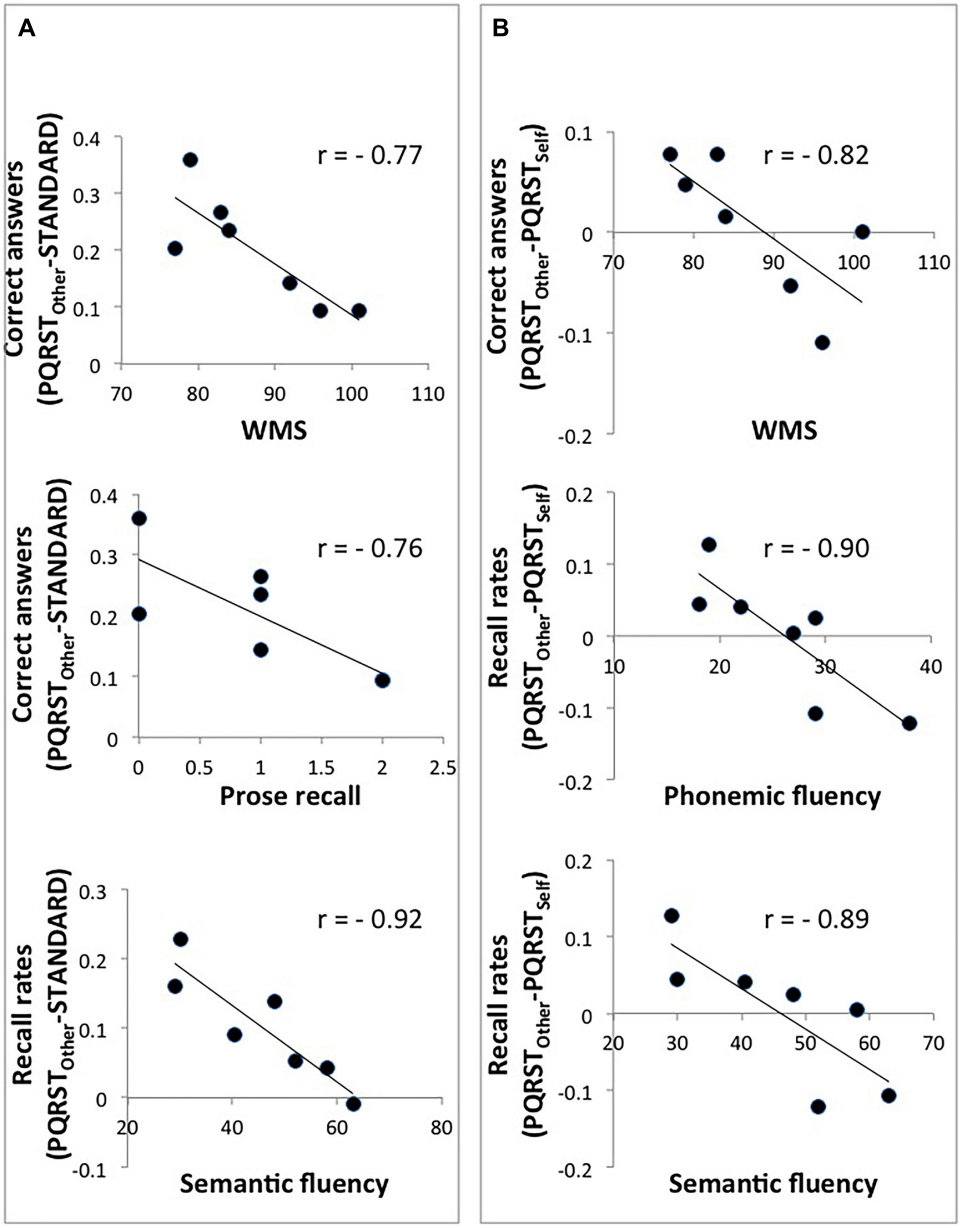
**Scatterplots of the correlations between patients’ scores at neuropsychological tests and performance differences between the PQRST-Other and the Standard condition **(A)**, and between the PQRST-Other and the PQRST-Self condition (B).** *p < 0.05.

We first investigated which aspect of patients’ neuropsychological profile predicted the degree of improvement in the PQRST-Other vs. Standard condition. We found that the improvement in answering questions about the texts in the PQRST-Other vs. Standard condition correlated negatively with standardized prose recall scores (*r*_Spearman_ = -0.76, *p* < 0.05) and WMS scores (*r*_Spearman_ = -0.77, *p* < 0.05), and the improvement in free recall correlated negatively with semantic fluency (*r*_Spearman_ = -0.92, *p* < 0.005; see **Figure 3A**). Thus, patients with weak memory and executive functioning benefited the most from a well-organized plan to encode new material. We next investigated which aspect of patients’ neuropsychological profile predicted performance differences between the PQRST-Other and PQRST-Self conditions. We found that differences in the ability to answer questions in the PQRST-Other vs. PQRST-Self condition correlated negatively with WMS scores (*r*_Spearman_ = -0.82, *p* < 0.05), and differences in free recall correlated negatively with semantic (*r*_Spearman_ = -0.89, *p* < 0.01) and phonemic fluency (*r*_Spearman_ = -0.90, *p* < 0.01; see **Figure 3B**). Thus, in individuals with more preserved memory and executive functioning the PQRST-Self condition tended to be as effective as the PQRST-Other condition.

## Discussion

The present study has two main findings. First, it shows that the PQRST method is effective in improving long-term memory in patients with mild memory and executive problems due to prefrontal cortex lesions. Specifically, the PQRST-Other condition, the version of the PQRST method most frequently described in the literature, led to an improvement in both the ability to answer questions about a text and in free recall, both immediately after study as well as after a delay. On average, patients exhibited a 28% improvement in the ability to answer questions and a 40% improvement in free recall. The second main finding of the study is that the same improvement was obtained in the PQRST-Self condition, a modified version of the PQRST procedure in which patients themselves formulated the questions to be used during the study and test phases, indicating that patients can benefit even from alternative forms of the PQRST method that load more heavily on self-initiated processes.

The present results are important in two ways. First, they show that internal methods aimed at optimizing memory encoding can improve memory performance significantly in patients with prefrontal lesions. Importantly, the improvement we observed was not limited to answering questions about a text, which could merely reflect encoding specificity, but extended to free recall, as well as to answering questions different from those that patients had considered at study (PQRST-Self condition, delayed test). This suggests that the PQRST method actually improved patients’ ability to encode and store new material.

It has often been hypothesized that patients with prefrontal cortex lesions exhibit problems in learning new information due to an impairment in engaging effective encoding strategies spontaneously (Moscovitch, 1992; Stuss et al., 1994; Gershberg and Shimamura, 1995; Shimamura, 1995; Alexander et al., 2009; Ptak et al., 2010). As discussed earlier, these patients may fail to select the relevant information to attend, and to process and organize it optimally for encoding. For example, an efficient strategy to learn lists of words, a task on which patients in the present study were highly impaired, is to associate them on the basis of their semantic relations, instead of repeating them passively. Making meaningful associations, and processing information semantically, however, require strategy selection and manipulation of information in working memory, which both depend on prefrontal cortex (Shallice and Burgess, 1991; Duncan and Owen, 2000; Baddeley, 2003; Nyberg et al., 2003). Savage et al. (2001) examined the neural bases of spontaneous and directed semantic organization strategies during verbal encoding and found that activity in the inferior prefrontal cortex, dorsolateral prefrontal cortex, and orbitofrontal cortex tracked the degree of semantic clustering observed in free recall. These regions may thus be crucial for the initiation of effective memory strategies (see also Schuck et al., 2015).

The systematic series of encoding operations probed by the PQRST method provided patients with a unique opportunity to process incoming information optimally for learning. Multiple mechanisms may underlie the efficacy of PQRST. First of all, PQRST favors deep (e.g., sematic) encoding of incoming information (Craik, 2002), requiring individuals to scrutinize and interpret the text carefully in order to answer the questions, and to participate actively in the learning process (e.g., Hunt and McDaniel, 1993). Moreover, the PQRST “forces” patients to use the questions as the structure around which they organize encoding. This may help patients to link the different parts of the story to each other, and to appreciate its meaning, again favoring semantic encoding. Notably, questions were available also in the Standard condition, but only the PQRST conditions explicitly demanded their usage. This aspect of the procedure is optimally suited for prefrontal patients, who fail in applying strategies spontaneously. Indeed, the more patients were impaired in memory and executive functions, the more they benefited from application of the PQRST. Another mechanism that may be responsible for memory improvements in the PQRST condition relates to the Question phase being a memory test. Recent research has shown that interpolating the study of prose passages with memory tests can substantially improve learning (Roediger and Butler, 2011), reducing lapses of attention (Pastötter et al., 2011) and mind-wandering (Szpunar et al., 2013). Several prefrontal cortex regions, including ventromedial prefrontal cortex (which was damaged in most our patients), are activated during mind-wandering (Christoff et al., 2009), and may help down-regulate mind-wandering during encoding.

In addition to the (classic) PQRST-Other condition, patients in the present study encoded texts through a modified PQRST-Self condition, in which they formulated the questions on their own. The rationale behind this choice was that, under these encoding instructions, patients would select the passages of the text that were relevant to them, motivating them to inspect the text carefully. Information that is more personally meaningful generally undergoes greater elaboration and organization at encoding, resulting in higher recall rates compared to information that does not have the same relevance (e.g., Klein and Kihlstrom, 1986; Symons and Johnson, 1997). Moreover, the PQRST-Self condition entails self-generation, another factor favoring learning (Slamecka and Graf, 1978). We did not find, however, an advantage of the PQRST-Self over the PQRST-Other condition. One possibility is that because the PQRST-Self condition is more demanding cognitively than the PQRST-Other condition, any advantage caused by self-relevant encoding is offset by the general reduction of cognitive resources in patients. Indeed, patients who performed relatively better in the PQRST-Self condition were those with more preserved executive functioning. Alternatively, given that the ventromedial prefrontal cortex is intimately related to self-processing (e.g., Philippi et al., 2012; D’Argembeau, 2013; Kim and Johnson, 2015), self-relevancy may have not played the strong role we expected in our patients. Interestingly, our data suggest an increased memory advantage from the PQRST-Self condition at the delayed compared to immediate test (i.e., in other-generated questions). Possibly, a brief delay supports the build-up of associations between different parts of the text and with existing semantic structures. Time-dependent memory consolidation effects are tipically found for emotional and rewarding material (Kensinger, 2009), as is self-relevant information. Future studies investigating the effect of different PQRST procedures at delays longer than 10 min, such as hours or days, would help clarify the possible mechanisms through which they operate. It would also be interesting to test the efficacy and neuropsychological basis of PQRST’s efficacy in healthy individuals. Unfortunately, a pilot study using our material evinced ceiling effects across conditions in these individuals.

The fact that the two PQRST procedures were equally effective in patients is of great importance for rehabilitation. As anticipated, the ultimate goal of rehabilitation is the ability to transfer the trained skills to other contexts than the laboratory, such as real life. The present finding that a PQRST procedure based on self-formulated questions is effective in ameliorating memory performance suggests that patients with prefrontal lesions could be trained to apply this method in real life, for example to keep track of news or to re-learn some aspects of their autobiography.

## Conclusion

We have confirmed that the PQRST is effective to promote new learning in patients with mild memory impairment, and shown that patients may benefit even from alternative versions of the procedure requiring higher levels of self-initiation. Future studies should verify whether patients can generalize the use of PQRST to untrained, and real-life situations. In our laboratory, we are currently investigating whether repeated encoding via the PQRST-Self procedure improves learning of untrained materials.

## Conflict of Interest Statement

The authors declare that the research was conducted in the absence of any commercial or financial relationships that could be construed as a potential conflict of interest.
